# A marketing perspective to “delight” the “patient 2.0”: new and challenging expectations for the healthcare provider

**DOI:** 10.1186/s12913-016-1285-x

**Published:** 2016-02-09

**Authors:** Luca Buccoliero, Elena Bellio, Maria Mazzola, Elisa Solinas

**Affiliations:** 1 Department of Marketing and CERMES (Centre For Research on Marketing and Services), Bocconi University, Via Roentgen, 1, 20136 Milan, Italy; 2 CERMES (Centre For Research on Marketing and Services), Bocconi University, Via Roentgen, 1, 20136 Milan, Italy

**Keywords:** Healthcare, ICTs, Empowerment, Patient 2.0, Healthcare Marketing Italy

## Abstract

**Background:**

The study aims at investigating the characteristics and the satisfaction determinants of the emerging patient profile. This profile appears to be more demanding and “empowered” compared to the ones traditionally conceived, asking for unconventional healthcare services and for a closer relationship with providers.

**Methods:**

Both qualitative (semi-structured interviews and focus groups) and quantitative (survey) analyses were performed on a random sample of 2808 Italian citizens-patients. Analyses entailed descriptive statistics, bivariate analysis and linear regressions.

**Results:**

Four relevant dimensions of patient 2.0 experience were identified through a literature review on experiential marketing in healthcare. Beta coefficients exhibited the effect that different healthcare experiential elements have on patient 2.0 satisfaction.

**Conclusions:**

Results allow to state that a new marketing approach, based on patient 2.0 characteristics and value drivers, should be adopted in the healthcare sector. Critical satisfaction drivers and new technological healthcare guidelines are identified in order to match the new patient profile needs.

## Background

The World Wide Web, social media contexts and technology advances (e.g., mobile devices) have turned around the way people communicate, broadening consumer ability to create and share product or service-related information [1]. Web 2.0 applications enable consumer empowerment [2] through increased connectivity (forums or communities) and an easier access to a large amount of information that make individuals increasingly aware and acknowledged about a brand or a product [3]. Variables conventionally pre-determined by firms, such as the exposure to product information or advertisements, are directly mastered by customers in the digital world [4]. Information and communication technologies (ICTs) suggest new and direct forms of interactions between customers and firms [5].

Indeed, the above-described emerging consumer behaviors developed also in the healthcare sector. Nowadays, new health service models arise from ICTs advances, failing the traditional concept that medical care must be provided in hospitals and be restricted to the sole patient-doctor relationship.

International studies have outlined how patient’s characteristics are changing over time. The adoption of ICTs in the healthcare sector has generated the so-called “Health 2.0”, enabling patient empowerment and education [6, 7]. “*Health 2.0 is the transition to personal and participatory healthcare. Everyone is invited to see what is happening in their own care and in the health care system in general, to add their ideas, and to make it better every day*”[8].

In this study, we refer to the new emerging patient type with the term “patient 2.0”.

The aim of this research is to deepen and develop the theme of “patient 2.0” in the light of the emergent ICTs developments in the healthcare sector. The study focuses on the state of diffusion and use, satisfaction and acceptability of ICTs in supporting patients.

To better understand the phenomenon of new behaviors and its implications for the healthcare sector, next section provides an overview on what this emerging patient’s profile refers to.

### Patient 2.0 seeks health related information online

Patients who look at the Internet as a healthcare tool are sometimes called “health seekers”, others “e-patients” [9–12]. E-patients use Internet to search for health related information [13], whether they are acting as patients or caregivers [10, 12]. The research drivers are wide-ranging: deepening the knowledge about one’s own health conditions and illness in a cost-effective way, getting a second opinion, especially when the information provided by the physician is not satisfactory, and, eventually, becoming more conscious about treatment options when talking to doctors [12].

The decision-making process about health and treatment choices of the above-mentioned patient profile differs from the one traditionally adopted.

Besides the learning experience, e-patients tend to trust the Internet tool. They use the information gathered for making important decisions regarding: the way they treat their illness or condition, whether to consult or not a doctor and, eventually, to get a second opinion [14]. Patients are frequently using websites to retrieve and provide information about doctors [10, 11].

As confirmed by the American Medical Association, an increasing number of people is seeking medical advice online, rather than actually visiting health professionals. When the search for medical information becomes compulsive, exceeding the frequency of search for sports, shopping or other topics, Internet users can be classified as “cyberchondriacs”. This term is used to identify a negative attitude of e-patients, characterized by an “*unfounded escalation of concerns about common symptomatology, based on the review of search results and literature on the Web*”. According to these users, getting the latest online medical information will positively affect the quality of consultation with the physician [15].

### Patient 2.0 wants to be involved in the healthcare process

The changing patient’s behaviors mostly affect the doctor-patient relationship. Patients engage in the healthcare process becoming less reliant on doctors as the sole source of information. Internet acts as an alternative source of information [16, 17] and fosters the phenomenon that many authors define as “patient empowerment” [18, 19]. Patients are “empowered” when they become more knowledgeable and responsible for their care: they assume an active role in the decision-making process and commit to treatment choices [19–21].

The new patient profile wants to actively monitor his/her health condition by accessing Personal Health Records (PHR) [11].

### Patient 2.0 is willing to accept health technology applications

The dynamics of communication between patients and doctors and among patients are changing. The increasing use of social media (e.g., social networks, community online, forum, blogs and so on) supports connections among patients [1, 22]: web networks allow to easily share personal experiences and to receive advices [11, 16, 23–26]. Furthermore, it is becoming increasingly common the practice of emailing care providers to advance enquiries about symptoms or to streamline general issues, related, for instance, to the cancellation of an appointment. E-patients seem to recognize the advantages of receiving medical assistance at distance and appear to be confident with the use of telemedicine devices and applications [11, 26].

### Patient 2.0 shows specific behavioral and socio-demographic characteristics

With reference to socio-demographic factors, literature allows to identify some specific clusters of patients 2.0 defined by gender, age and education [10, 12], while there is no significant evidence of the effect of the household income on behaviors [12, 27].

There is some indication that women, young adults and well-educated people are most likely to seek online health information [10, 12, 27]. Female internet users are more likely to participate to patient support groups [28, 29] and they use virtual communities mostly to share personal experiences and to give encouragement, rather than to only gather or give information as men do [29].

Moreover, young adults perceive more beneficial the opportunity to access medical records online [10]. Finally, at increasing education levels potentially correspond higher rates of innovation adoption [11].

In conclusion, it can be stated that a new paradigm of healthcare service is emerging, arising from a different demand, which is continually subject to ongoing innovations and technologies.

### Patient 2.0 wants to live a quality experience in care environments

Many authors have identified as sources of patient satisfaction the following main categories of value elements:

- Atmosphere & Comfort: factors related to the hospital environment, such as colors, lighting, smell and hygiene, food quality, noise and so on [30–38];
- Patient empowerment: the inclusion of patients in the caring process as a result of the provision of more detailed information by health care professionals; patients become active participants in the decision-making process [33, 36, 39–53];
- Privacy & Dignity: the necessity to treat patients with respect, hence, considering them as individuals and not simply service users [34, 35, 54–58];
- Technology: health technology applications shape patients’ experience. This element refers to available ICTs which maximized patients access to healthcare services [58–63].

Literature analysis suggests some gaps and limitations in current studies about the topic.

Firstly, detailed definitions of “patient 2.0” are currently hard to find in the available scientific literature. Whereas health 2.0 has been widely discussed in previous studies and despite some authors tried to identify the characteristics and attitudes of this profile, there is a lack of specific knowledge about its satisfaction drivers and traits. A great attention has been paid to health informatics advances and their economic benefits in the healthcare market [21], while less focus has been addressed to patient’s behaviors and compliance to those new technologies. Only some indications about specific determinants of patient satisfaction have been assessed in each study. Furthermore, there are only few lessons that can be generalized since analysis are normally focused on specific health issues.

## Methods

**Fig. 1:**
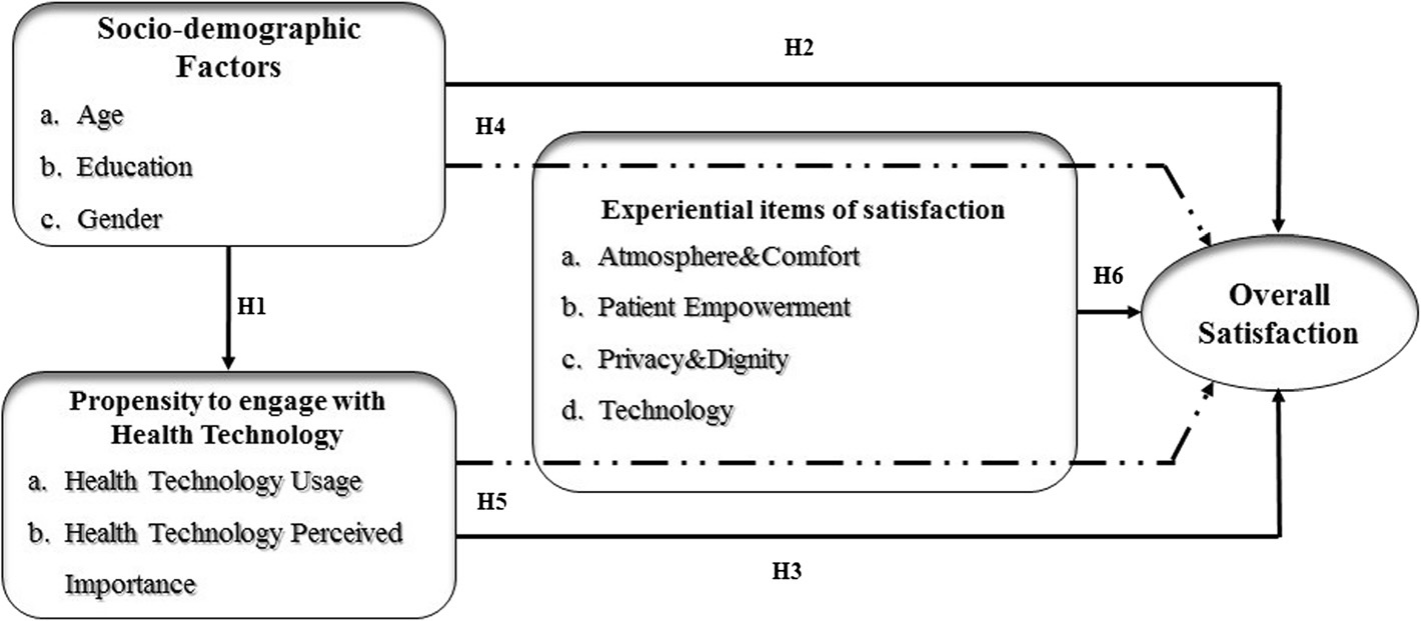
Research Framework for Patient 2.0 Phenomenon Analysis

This work aims at understanding patient 2.0 satisfaction drivers by considering and testing together different items as available in current literature which are linked to: socio-demographic factors, propensity to engage with health technologies and dimensions that shape the experience (as shown in Fig. 1).

Through this research it will be also possible to identify the main value proposition items on which healthcare providers should focus in order to maximize the effectiveness of their “patient 2.0-centered” approach.

The following research hypotheses were tested:

- H1. Socio-demographic factors (a. age, b. education, c. gender) are related to patient’s propensity to engage with health technology (a. health technology usage, b. health technology perceived importance).
- H2. Socio-demographic factors (a. age, b. education, c. gender) are related to overall satisfaction.
- H3. Propensity to engage with health technology (a. health technology usage, b. health technology perceived importance) is related to patient overall satisfaction.
- H4. Overall satisfaction is different according to socio-demographic factors, and is influenced by the degree of satisfaction within each experiential items (a. Atmosphere&Comfort, b. Patient Empowerment, c. Privacy&Dignity, d. Technology).
- H5. Overall satisfaction is different according to Propensity to engage with health technology (a. health technology usage, b. health technology perceived importance), and is influenced by the degree of satisfaction within each experiential items (a. Atmosphere&Comfort, b. Patient Empowerment, c. Privacy&Dignity, d. Technology).
- H6. Patient experiential items of satisfaction are related to overall satisfaction.

After a deep literature review, a qualitative research phase started. Semi-structured interviews to ICTs experts and healthcare providers were conducted and three focus groups involving from six to eight citizens-patients each were performed to understand the phenomenon from both health providers and patients sides. The software NVivo [64] was used as research tool to glean insights and develop meaningful and evidence-based conclusions.

Those results helped to better structure the quantitative research that was later conducted on a sample of 2808 citizens-patients of the Niguarda Ca’ Granda hospital in Milan-Italy. Niguarda Hospital offers a full range of clinical and surgical specialties and is acknowledged in Italy as one of the main transplant organizations with 26 specialized national referral centers and 4124 employees in total. The structure is equipped with 1213 beds (1138 ordinary and 75 day hospital), 34 operating theatres and 340 ambulatory rooms and, last year, dealt with 40465 admissions and 91896 ER admissions. Since its foundation in 1939, the hospital seeks the excellence by continuous improvements through investments in latest healthcare technologies and the promotion of health cooperation with various international organizations. Thanks to this strategy, Niguarda Hospital has built its strong brand reputation.

**Fig. 2:**
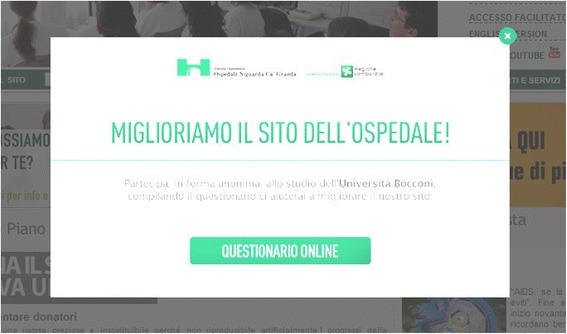
Online survey – Pop up image

The quantitative analysis was performed by administering: a paper-based questionnaire to 737 outpatients and 861 inpatients (i.e., offline sample) from October to December 2013. For what concerns inpatients, data were collected in the following departments: intensive care, internal medicine, obstetrics and gynecology. Outpatients, instead, were recruited in the day-hospital department and in the obstetrics and gynecology clinic. We decided to consider these hospital departments, as we wanted to avoid contacting patients with deficit that could affect their ability to properly answer our questions. The questionnaires were administered by hospital staff, which usually manage the customer care section, after been instructed by the authors. Patients were randomly chosen among those willing to collaborate. More in details, patients were free to decide whether to collaborate or not in the study and the opportunity was made available to all inpatients in the mentioned departments in the time period considered. In addition, an online survey was also administered to 1210 users of the institutional hospital website (i.e., online sample) from January to May 2014. The online questionnaire appeared as a pop up in the hospital website homepage (see Fig. 2) and each user could freely decide whether or not to collaborate by answering the survey.

The questionnaires were administered in Italian and both socio-demographic information and health technology acceptance information (extent of use of IT tools and social media to manage health related issues) were gathered. A specific section also assessed patients’ satisfaction with health services, with ICT based tools and touch points and with the “overall” experience of care. Responses were based on a Likert scale ranging from one to seven (where the successive Likert category represents a “better” response than the preceding value), dichotomous and checklist items.

Statistical Package for the Social Sciences Program (SPSS) version 21 was used for the statistical analysis. An early investigation of the sample composition was made through descriptive statistics. A Chi-squared test was used to assess the association between different categorical variables. Pearson correlation coefficients were calculated to measure the degree of linear dependence between satisfaction variables. The analysis of variance (ANOVA) was used to infer whether there are real differences between the means of independent groups in the sample data. Eta-squared values from ANOVA were reported as a measure of effect size for group mean differences [65]. Patient overall satisfaction was investigated by analyzing the level of patient overall service satisfaction. This information was obtained through one Likert item question. Furthermore, satisfaction for each experiential item (atmosphere and comfort, patient empowerment, privacy and dignity, and technology) was also measured using the same Likert scale. Then, linear regressions were used to assess the impact of each experiential items of satisfaction (independent variables) on overall satisfaction (dependent variable). Regression analysis were performed in the light of various socio-demographic variables to determine whether variances regarding experiential items of satisfaction exist for different patient population subsets. The statistical significance was defined as *p*-value lower than 0.05 and all the β coefficients reported in this study were statistically significant.

## Results and discussion

### Qualitative analysis

**Fig. 3:**
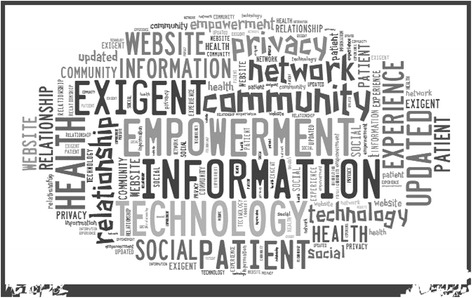
Content Analysis – Tag cloud of main insights

Qualitative content analysis was performed. A tag cloud (see Fig. 3) was used as visualization tool in order to sum up and emphasize the most frequently occurring words and main insights, resulting from both focus groups and semi-structured interviews [66–68]. The tag cloud outlined four words with larger font sizes: *exigent, empowerment, information* and *technology*. Those, as expected, are used with a different meaning and degree of acceptance since the perspectives of experts and patients differ. Patients believe they should obtain a higher degree of *empowerment* and an active role in the care process, thanks to *technology* and the deriving unlimited *information* that becomes available. Experts, on the other hand, recognize the *empowerment* phenomenon and the emerging challenge to face a more demanding patient and underline the opportunity to meet those patients’ needs by a proper use of technology.

### Quantitative analysis - Sample description

The average age of the sample is 49 years (youngest respondent 16 y.o and oldest 98 y.o). Females represent 52 % of the sample. Age was categorized in four main groups following European Union categorization: patients from 18 y.o. to 25 y.o (6 % of sample, c. 108 respondents), patients aged 26–45 y.o. (34 % of sample, c. 600 respondents), patients aged 46 y.o. till 65 (40 % of sample, c. 705 respondents) and, eventually, patients over 65 y.o. (21 % of sample, c. 372 respondents). Total missing answers to the age question were more than 1000.

**Fig. 4:**
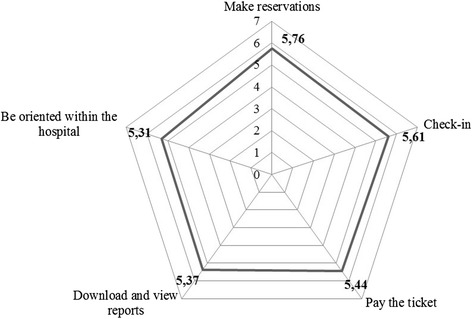
Mean values of importance for mobile health applications services

Mobile health applications (mHealth apps) are of undoubted importance for patients as shown in Fig. 4, which describes the five main activities performed through mobile phones and their relative levels of importance.

**Fig. 5:**
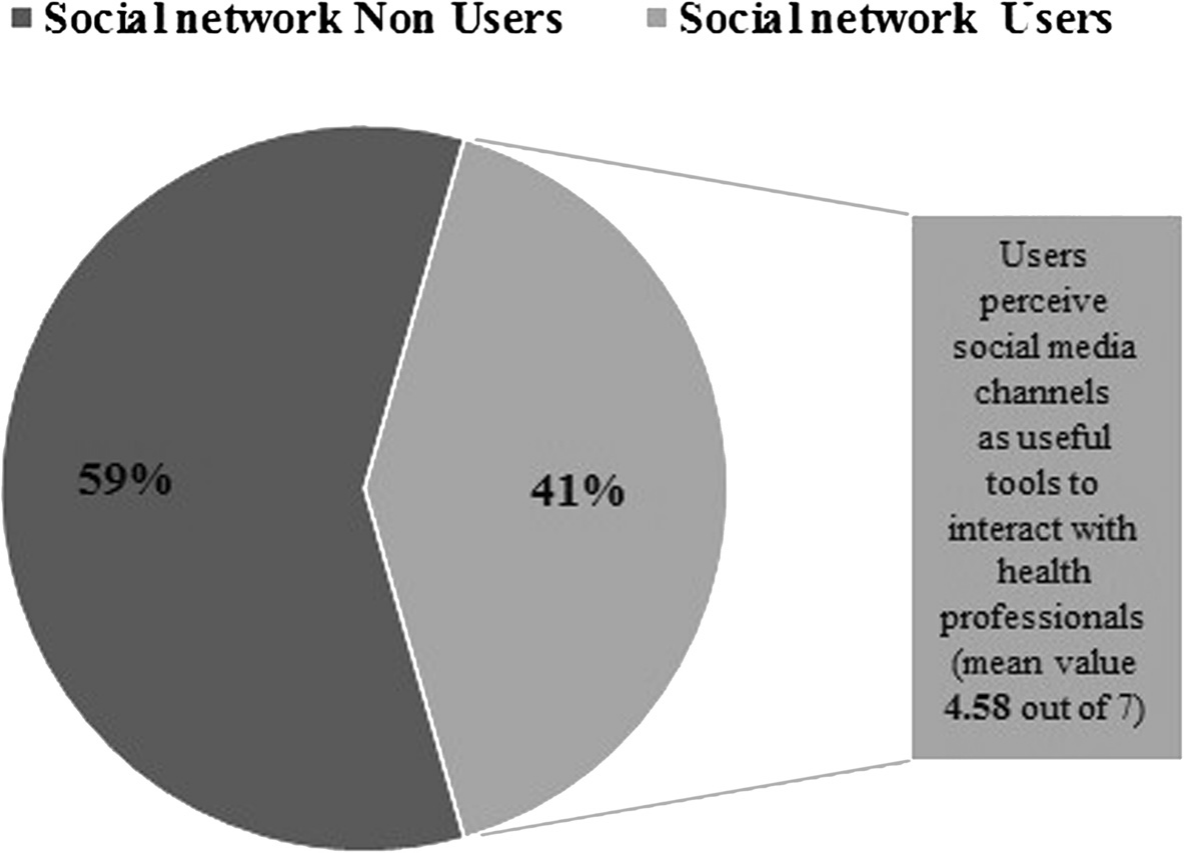
Social Network usage and perceived usefulness of the hospital presence on social media channels

Social networks are used by 41 % of the sample and users declare to highly appreciate the active presence of the hospital on social media (see Fig. 5).

Respondents’ main reason to access the hospital website is the search for health and administrative information. There is a great extent of use of ICTs to carry out health related issues: 63 % of patients use the regional smartcard issued by Lombardia Region to access their medical records.

The Research hypotheses, earlier formulated, guided the study below. The main significant statistical results are presented to provide evidences that identify the value items that mostly affect “patients 2.0” satisfaction.

### Propensity to engage with health related technology (H1)

Socio-demographic factors were used to detect segments that behave differently with reference to the propensity to engage with health technology applications.

As for health technology usage, age and level of education appear to be discriminant factors with regard to the frequencies observed in Pearson’s Chi-squared test (see Table 1). There is a statistically significant association between variables (5 % significance level), as confirmed by Phi and Cramer's V values higher than 0.2.

**Table 1:** Pearson’s Chi-squared test. Health technology usage - observed frequencies

Discriminating factor	Web site (percentage of users)	PHR (percentage of users)
Age	60 % people aged 25–45 y.o.	55 % people aged 25–45 y.o.
Phi and Cramer’s V values	0.272	no significant relationship
Education level	72 % medium and high	53 % medium and high
Phi and Cramer’s V values	0.264	no significant relationship

As for the health technology applications perceived importance, mean values strongly change if considering age, education level, gender and social network usage (see Table 2). Eta-squared values of 0.3 show that variables are strongly associated.

**Table 2:** Analysis of variance (ANOVA). Mean values of perceived importance – differences between group means

	Discriminating factor
Mhealth apps	Age: People aged 18-25: 6 Vs. Over 65: 4.80 Education level: Higher: 5.9 Vs. Lower: 4.6 Social network users: Users: 5.83 Vs. Non Users: 4.85
Communication 2.0 (email)	Social network users: Users: 5.5 Vs. Non Users: 4.6
Online health communities	Online health communities: Female: 5.51 Vs. Male: 4.80
Hospital on social media	Hospital on social media: Users: 4.58 Vs. Non Users: 3.89

### Patient overall satisfaction (H2, H3)

The total sample claimed to be satisfied with their overall experience at the Niguarda hospital. The level of overall satisfaction is on average high for the whole sample and also for each of the three patients’ groups, as follows: inpatients (6.04 out of 7), on line respondents (6 out of 7) and outpatients (5.65 out of 7).

Pearson correlation coefficients show positive and significant correlations at the 0.01 level. The amount of information received is strongly correlated with the overall satisfaction (ρ: 0.682) and the perceived quality of cure (ρ: 0.675).

Young patients (aged 30–45) show lower levels of overall satisfaction in comparison with older people (mean = 4.86/7 for people aged 18–25 against 5.78 for over 65 respondents). Eta-squared value is equal to 0.34, indicating a strong association between variables. The satisfaction scores related to graduated patients confirm this phenomenon: they are less satisfied of the overall experience (mean = 5.76 against 6.15 of those with lower education). As regards the information received, women are harder to please compared to man (mean = 5.60/7 against 5.91/7 of the male sample and Eta-squared equal to 0.20).

### Patient experiential items of satisfaction (H4, H5, H6)

Patient satisfaction determinants were detected by analyzing the impact of each experiential item of satisfaction (independent variables) on overall patient satisfaction (dependent variable) through linear regression analysis. The experiential items are derived in this study through an aggregation of various questionnaire items intended to measure the corresponding above construct (experiential item).

Cronbach’s alpha coefficients were performed to test the reliability of aggregated items. Test scores exhibit a good internal consistency reliability (alpha values higher than 0.6) [69].

**Fig. 6:**
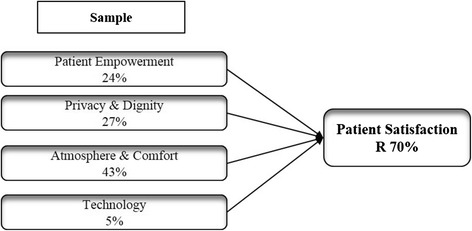
Experiential items of satisfaction - β standardized coefficients for the offline sample

**Fig. 7:**
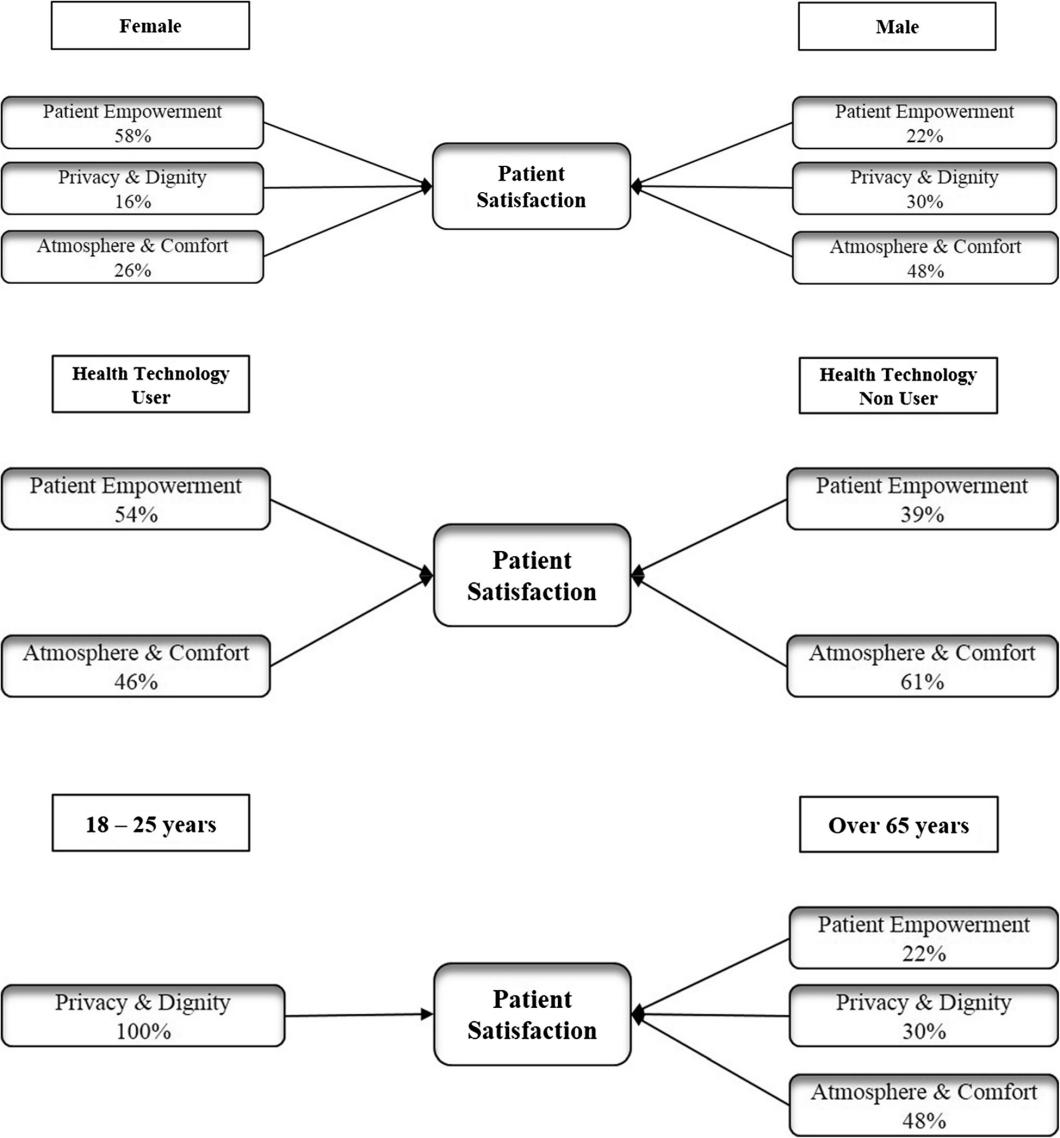
β standardized coefficients for the sample subsets

Figure 6 shows the impact of each experiential item on the overall satisfaction reporting β coefficients derived from a regression analysis. Figure 7 illustrates how the effect on the overall satisfaction determined by each experiential item changes accordingly to socio-demographic and behavioral characteristics of patients.

Table 3 displays a summary of results coming from the quantitative analysis.

**Table 3:** Hypotheses test - Summary of results

Hypotheses	Results	Statistical Analysis
H1aa: Age is related to Health Technology Usage.	H1aa: Observed frequencies of technology users are higher in the segment of people aged 18–25 y.o.	Pearson’s Chi-squared test
H1ba: Gender is related to Health Technology Usage.	H1ba: No significant differences by gender in technology usage.	
H1ca: Education level is positively related to Health Technology Usage.	H1ca: The greatest percentage of technology users is found in the segment of people with higher level of education.	
ᅟ	ᅟ	
H1ab: Age is negatively related to Health Technology Perceived Importance	H1ab: The importance of health technology is higher for younger people	Analysis of Variance (ANOVA)
H1bb: Gender is related to Health Technology Perceived Importance	H1bb: Women, compared with men, perceive as more important getting health support via web.	
H1cb: Education level is positively related to Health Technology Perceived Importance.	H1cb: Unlike people with no education, well-educated patients consider technologies relevant in the health sector.	
ᅟ	ᅟ	
H2a: Age is positively related to Overall Satisfaction.	H2a: Young adults are less satisfied with the service received than over 65 people.	Analysis of Variance (ANOVA)
H2b: Gender is related to Overall Satisfaction.	H2b: Women are not as satisfied as men regarding the overall service.	
H2c: Education level is negatively related to Overall Satisfaction.	H2c: At higher educational levels, lower levels of overall satisfaction correspond.	
ᅟ	ᅟ	
H3a: Health technology usage is related to Overall Satisfaction.	No significant differences by Health technology usage and the related attached importance in levels of overall satisfaction.	Analysis of Variance (ANOVA)
H3b: Health technology perceived importance is negatively related to Overall Satisfaction.		
ᅟ	ᅟ	
H4aa: Age is related to Atmosphere&Comfort.	H4aa, H4ab, H4ac: Age is related to Atmosphere&Comfort, Patient Empowerment and Privacy&Dignity. These experiential items of satisfaction are positively related to Overall Satisfaction for the sole segment of over 65 patients.	Regression Analysis
H4ab: Age is related to Patient Empowerment.		
H4ac: Age is related to Privacy&Dignity.		
	Atmosphere&Comfort has the greatest impact on Overall Satisfaction (47%), followed by Privacy&Dignity (30%) and finally Patient Empowerment (22%). For the youngest segment only Privacy&Dignity proved to have an impact on the Overall Satisfaction.	
H4ba: Gender is related to Atmosphere&Comfort.	H4ba, H4bb, H4bc: Gender is related to these Experiential items of Satisfaction. Patient Empowerment affects more than other items the Overall Satisfaction of female patients (58%), while for men Atmosphere&Comfort has the greatest impact on satisfaction (48%).	
H4bb: Gender is related to Patient Empowerment.		
H4bc: Gender is related to Privacy&Dignity.		
H4ad: Age is related to Technology.	H4ad, H4bd, H4ca, H4cb, H4cc, H4dd: No statistically significant relationships between Age, Gender and Technology and the Education level and all the Experiential items of Satisfaction.	
H4bd: Gender is related to Technology		
H4ca: Education level is related to Atmosphere&Comfort.		
H4cb: Education level is related to Patient Empowerment		
H4cc: Education level is related to Privacy&Dignity.		
H4dd: Education level is related to Technology.		
ᅟ	ᅟ	
H5aa: Health technology usage is related to Atmosphere&Comfort.	H5aa, H5ab: Health technology usage is related to Atmosphere&Comfort and Patient Empowerment. Technology users appear to be more satisfied with the overall service when empowered. While no users consider hospital environment characteristics as fundamental parameters of the care experience evaluation.	Regression Analysis
H5ab: Health technology usage is related to Patient Empowerment.		
H5ac: Health technology usage is related to Privacy&Dignity.	H5ac, H5ad: No statistical evidence about these relationships	
H5ad: Health technology usage is related to Technology.		
H5ba: Health technology perceived importance is related to Atmosphere&Comfort	H5ba, H5bb, H5bc, H5bd: No statistically significant relationships between Health Technology importance and the experiential items of satisfaction.	
H5bb: Health technology perceived importance is related to Patient Empowerment.		
H5bc: Health technology perceived importance is related to Privacy&Dignity.		
H5bd: Health technology perceived importance is related to Technology.		
ᅟ	ᅟ	
H6a: Atmosphere&Comfort is positively related to Overall Satisfaction.	(a) All the Experiential items of satisfaction affect the level of overall satisfaction. They impact on satisfaction in the following order:	(a) Regression Analysis
		(b) Correlation Analysis
H6b: Patient Empowerment is positively related to Overall Satisfaction.	Atmosphere&Comfort (43%).	
	Privacy&Dignity (27%).	
	Patient Empowerment (24%).	
	Technology (5%).	
H6c: Privacy&Dignity is positively related to Overall Satisfaction.	(b) Patient Empowerment is positively correlated with the Overall Satisfaction. It emerges that when patients are accurately informed (consequently empowered) by physicians, they become more satisfied too.	
H6d: Technology is positively related to Overall Satisfaction.		

## Conclusion

The qualitative analysis underlined that healthcare organizations are not fully developed according to patients’ emerging needs. Experts underline that information on webpages are usually not updated, resulting useless for patients. According to them, technology is a facilitator in everyday life, especially for younger people. Therefore, since the increasing technology acceptance, care providers should take advantage of the opportunities offered by ICT applications to simplify processes and bureaucracy. Focus group participants report that many of their questions to doctors go unanswered. The need of more detailed information is arising. Patients affirm that they usually rely on IT tools in order to fill their information needs.

The quantitative analyses identify patient segments that behave differently with regard to the different tested hypotheses.

The propensity to engage with health related technology (H1) results to be higher among younger and well-educated people. Table 1 illustrates that the usage of PHR and hospital website appears to be more widespread among younger people and respondents that own an academic degree. Table 2 shows in details how mobile health applications are more relevant for younger, well-educated people and social media users. In addition, female respondents appreciate more than male ones the opportunity to get online support from care providers and patients. They are also more likely to share their experience with others using the virtual community tool.

With regard to patients’ overall satisfaction (H2, H3), the one-way ANOVA returned interesting results: gender, age and the education level differentiate the degree of satisfaction among different population sub-groups. More specifically, women, young respondents and those with a higher education (high school or undergraduate) appear to be less satisfied and more demanding with reference to the service received.

Finally, if considering patient experiential items of satisfaction (H4, H5, H6), the greatest impact on overall satisfaction is determined by elements related to environmental characteristics of the hospital. Further analyses on specific sample segments show how the effect on the overall satisfaction determined by each experiential item varies depending on socio-demographic and behavioral characteristics of patients (see Fig. 6). Patient Empowerment, for instance, plays a significant role in determining satisfaction in female patients and health technology users. Hence, to delight those patient segments, physicians are asked to provide them more detailed information, so allow them to participate in the decision-making process and consequently better manage health conditions.

With regard to youngest patients, Privacy&Dignity proved to be the sole relevant experiential item of satisfaction.

There is a need to identify the critical dimensions of the healthcare patients’ experience. To our knowledge, there is no previous research that focuses on determining which are the most valued health experiential elements considering patients emerging requests. Available researches were predominantly focused on patient experience and satisfaction analyses distinguishing patients by disease. In this study, we attempt to provide a cross-disease identikit of the emerging patient profile and a comprehensive investigation of the critical patient experience dimensions. Measuring the effect of patient experience dimensions on overall satisfaction, we investigate to what extent patient satisfaction is affected by each of them. Once we know which dimension mostly drives satisfaction, it becomes easier to develop specific policies that match the health quality requirements of “patient 2.0”.

In conclusion, results provide evidence that a new patient type, defined in this study as “patient 2.0”, emerges in today’s target population. This profile mainly deals with a young and well-educated person, increasingly confident with the use of technology to carry out health related activities. The “patient 2.0” is willing to accept ICT health applications and to switch to new and unconventional service delivery solutions in order to get a faster and better service.

Moreover, this study is intended to fill the gap in current studies about patient 2.0 value items: patients seem to be more satisfied with their medical experience when healthcare providers treat them with respect, accurately managing their personal data, providing more information about their health, enabling patient empowerment.

Finally, digital communication channels as emails or virtual communities are perceived by the “patient 2.0” as an effective way to get support and information.

The main strength of this study was looking at hospital performance from patients' perspective rather than from the health care provider’s point of view.

Results allow to state that matching patient 2.0 value drivers requires new compelling value propositions of health providers based on the following assumptions:

- Providing patients with more clear and detailed information to let them make informed choices about treatments and take control of their health care needs;
- Implementing a multichannel communication strategy to facilitate interactions between physicians and patients and to support patients via web tools (dedicated online web platforms, emails, and so on);
- Paying more attention to patient data management and privacy: treating patients as individuals by directing care providers’ attention to patient data protection and respect;
- Maximizing health service delivery: streamlining health operations via the integration of ICTs into the health care system (Mhealth and telemedicine applications).

The above-described objectives should inspire a new marketing approach in the healthcare sector, since the technological acceptance trend appears to be widespread among all age groups, although the youngest patients are the main exponents of this phenomenon.

A cultural change is required too: new managerial figures to support and improve patient experience, such as the Chief Consumer Officer (CCO) or the Chief Experience Officer (CXO) [70], should be introduced also in the healthcare sector, as it is happening in other industries [71].

This current study could be further developed by benchmarking experiential dimensions across hospitals and patients with different demographic and health profiles in order to observe if real dissimilarities exist. Furthermore, it could be tested if individual health technology acceptance depends on both patient and hospital characteristics or may be also affected by expectancies, previous subjective experiences and the technological level of the analyzed context.

### Ethics and consent

The authors declare that, given the nature of collected and analyzed data, no ethic authorizations and no consent declarations were required by Niguarda Hospital Board of Directors, who authorized data collection and research publication according to the Italian Law.

Italian National Health Service (Legislative details: Law N.211 24/06/2003, Ministry of Health decree N.45 08/02/2013, Lombardia Region decree N.5493 25/06/2013) requires the preliminary ethical approval of research only in case of clinical trials of investigational medicinal products, medical devices, drug/device combination, clinical investigation. Our study is solely a service/satisfaction evaluation.

Furthermore, Italian law on privacy and data protection (Law N. 196/2003) requires formal actions just in case of personal and/or sensitive data. Our questionnaires are completely anonymous and no personal information are linked or linkable to a specific respondent. Moreover, given the nature of the study, no sensitive data was collected or analyzed.

### Availability of supporting data

Dataset are available in SPSS format upon request to the corresponding Author.
